# Clinical analysis of sixty-nine children with anomalous aortic origin of the coronary artery

**DOI:** 10.1007/s00431-023-05075-0

**Published:** 2023-07-12

**Authors:** Zhen Zhen, Ziyan Dong, Jia Na, Xi Chen, Qirui Li, Lu Gao, Yue Yuan

**Affiliations:** Department of Cardiology, Beijing Children’s Hospital, Capital Medical University, National Centre for Children’s Health, 56 South Lishi Road, Beijing, 100045 China

**Keywords:** Anomalous aortic origin of the coronary artery, Children, CT coronary angiography, Clinical presentation, Anatomy

## Abstract

We aimed to analyse the clinical characteristics of children with different types of anomalous aortic origin of the coronary artery (AAOCA) at different ages, and to discuss the factors related to myocardial ischaemia. In this retrospective study, we included 69 children diagnosed with AAOCA using CT coronary angiography; we classified the participants based on the type of AAOCA, age, and high-risk anatomy. The clinical characteristics of the different AAOCA types and age groups were compared, and the correlation between manifestations and high-risk anatomy was analysed. Anomalous origin of the left coronary artery from the right coronary artery sinus, anomalous origin of the right coronary artery from the left coronary artery sinus, and a coronary artery origin without coronary sinuses was found in 10 (14.5%), 57 (82.6%), 2 (2.9%) patients, respectively. There were no significant differences in sex, clinical manifestations, percentage of positive myocardial injury markers, electrocardiogram, transthoracic echocardiography, or proportion of high-risk anatomy among the groups with different AAOCA types. According to age group, the proportion of asymptomatic infants and pre-schoolers was the highest (p < 0.001). Forty-three patients (62.3%) had high-risk anatomy and were more likely to present with severe symptoms and cardiac syncope (p < 0.05).

*Conclusion*: There were no significant differences in the proportions of high-risk anatomy and clinical characteristics among children with different AAOCA types. We found a relation between the severity of AAOCA clinical symptoms and anatomical risk.

**Table:** 

**What is Known:** *• Clinical symptoms in children with AAOCA are varied and the results of routine cardiological examinations lack specificity.* *• High-risk anatomical features, exercise, cardiac symptoms, and ALCA are risk factors for the occurrence of SCD in patients with AAOCA.*	
**What is New:** *• Compared the clinical characteristics of different types of AAOCA and ages.* *• Analysed the correlation between symptoms and high-risk anatomical features.*

**What is Known:**

*• Clinical symptoms in children with AAOCA are varied and the results of routine cardiological examinations lack specificity.*

*• High-risk anatomical features, exercise, cardiac symptoms, and ALCA are risk factors for the occurrence of SCD in patients with AAOCA.*

**What is New:**

*• Compared the clinical characteristics of different types of AAOCA and ages.*

*• Analysed the correlation between symptoms and high-risk anatomical features.*

## Introduction

Anomalous aortic origin of the coronary artery (AAOCA) is a rare congenital heart disease in which one (or rarely two) of the coronary arteries originates from the wrong aortic sinus. In AAOCA, vessels mainly originate from the contralateral coronary sinus, including the anomalous origin of the left coronary artery from the right coronary artery sinus (ALCA) and the right coronary artery from the left coronary artery sinus (ARCA). Very rarely, the left or right coronary arteries originate from the non-coronary sinus. Clinical symptoms in children with AAOCA are varied and the results of routine cardiological examinations, such as myocardial damage markers, electrocardiography (ECG), and transthoracic echocardiography (TTE), lack specificity. TTE is the preferred technique for initial identification of AAOCA in children, whereas CT coronary angiography (CTCA) is the main diagnostic method. Studies have shown that AAOCA is the second leading cause of sudden cardiac death (SCD) in athletes [1]. According to the American College of Cardiology/American Heart Association (AHA/ACC) guidelines [2], high-risk anatomical features, exercise, cardiac symptoms (syncopation and chest tightness), and ALCA are risk factors for the occurrence of SCD in patients with AAOCA. Our retrospective study evaluated 69 children with AAOCA, compared the clinical characteristics of different types of AAOCA and ages, and analysed the correlation between symptoms and high-risk anatomical features.

## Materials and methods

### Study participants

This was a retrospective study of 69 children diagnosed with AAOCA using CTCA from January 1st, 2014, to December 31st, 2022 at Beijing Children's Hospital Department of Cardiology. Our study was approved by the Medical Ethics Committee of Beijing Children's Hospital, Capital Medical University (approval number: [2023]-E-021-R) and informed consent was signed by all children’s guardians.

### Inclusion and exclusion criteria

Inclusion criteria: 1) patients aged < 18 years and 2) diagnosed with AAOCA (ALCA, ARCA, or coronary artery originating from the non-coronary sinus) by CTCA or surgery.

Exclusion criteria: 1) patients with complex cardiac malformations (such as tetralogy of Fallot, transposition of the great arteries, and pulmonary atresia) associated with AAOCA and 2) patients with acquired coronary artery disease.

### Study methods

All children admitted to our hospital with cardiac symptoms were routinely examined using myocardial damage markers, ECG and TTE. If there was evidence of myocardial injury or myocardial ischemia and suspected coronary artery abnormality, CTCA was performed to confirm the diagnosis. For children diagnosed with AAOCA, clinical data including age, sex, symptoms, myocardial damage markers, ECG, imaging features (TTE and CTCA), treatment regimen, and follow-up results were reviewed.

A VCT64 Discovery CT750HD scanner (GE Healthcare, Chicago, IL, USA) was used to diagnose AAOCA by reconstructing images of the coronary, aortic, and pulmonary arteries. A Philips IE33 colour Doppler TTE (Amsterdam, Netherlands) was used to investigate the cardiac structure, origin, and course of the coronary arteries, and cardiac function. Decreased cardiac function was defined as left ventricular ejection fraction (LVEF) ≤ 55%. Myocardial damage marker (creatine kinase myocardial band [CK-MB], troponin I [TnI], brain natriuretic peptide [BNP], and N-terminal pro-B-type natriuretic peptide [NT-proBNP]) positivity was defined as ≥ 1 index higher than normal levels (Table 1). ECG changes were divided into five categories [3]: 1) myocardial ischaemic changes (ST-T changes/pathological Q-wave); 2) conduction system damage (atrioventricular block/bundle branch block); 3) sinus node dysfunction (sinus bradycardia/sinus arrest/sinus atrial block); 4) sinus tachycardia; 5) pre-stage contraction (atrial premature beat/ventricular premature beat). Patients who underwent surgery had follow-up appointments at 1, 3, 6, and 12 months after discharge from the hospital. Other patients were followed up according to their conditions. All patients were contacted and asked if any cardiovascular events (SCD, myocardial infarction, coronary revascularisation or hospitalisation due to disease) had occurred after discharge.

**Table 1:** Standard levels of myocardial damage markers assessed in our study

Myocardial damage markers	Normal level
CK-MB	0–25 U/L
TnI	0.000–0.026 ng/mL
BNP	0.00–100.00 pg/mL
NT-proBNP	< 450 pg/mL

*BNP* brain natriuretic peptide, *CK-MB* creatine kinase myocardial band, *NT-proBNP* N-terminal pro-B-type natriuretic peptide, *TnI* troponin I

### Grouping method and definitions

According to the AAOCA type, patients were stratified into three categories based on CTCA or surgical results: ALCA, ARCA, and coronary artery originating from the non-coronary sinus. To analyse the clinical characteristics of different age groups, we categorised patients into infant and toddler (< 3 years old), preschool (3–5 years old) and school age groups (≥ 6 years old). Patients were categorised into high-risk and low-risk anatomy groups; high-risk anatomical features included coronary artery orifice stenosis or a slit-like orifice, an inter-arterial course, an acute take-off angle, or an intra-mural course.

Orifice stenosis or slit-like orifice: The area and two orthogonal diameters of the anomalous coronary artery orifices were measured. Half the distance between the anomalous artery origin and its return to the correct anatomical position was used as the reference area to identify orifice stenosis. A slit-like orifice is defined as a coronary artery orifice with a maximum diameter greater than twice the orthogonal measurement value.

Inter-arterial course: Right anterior oblique CTCA was used to visualise a cross-section of the coronary artery in the AAOCA between the aorta and pulmonary artery. A vertical-to-horizontal ratio of coronary artery diameter more than 2:1 is indicative of significant stenosis (Cheong-Angelini sign), which was used to indicate an inter-arterial course.

Acute take-off angle: The angle between the line parallel to the coronary sinus wall and the line parallel to the proximal course of the coronary artery was measured in the plane parallel to the aortic annulus. If the angle was < 45 °, it was defined as an acute take-off angle.

Intra-mural course: The intra-mural length was measured as the distance from the orifice of the AAOCA to its point of exit from the aortic wall.

### Statistical analysis

Statistical analysis was performed using SPSS 26.0 software (IBM Corp., Armonk, NY, USA). Results are presented as mean ± standard deviation for continuous variables, and as numbers (percentages) for categorical data. Chi-squared, corrected chi-squared, and Fisher’s exact tests were used to compare categorical variables. A two-sided p value < 0.05 was considered statistically significant.

## Results

### Baseline characteristics

**Fig. 1:**
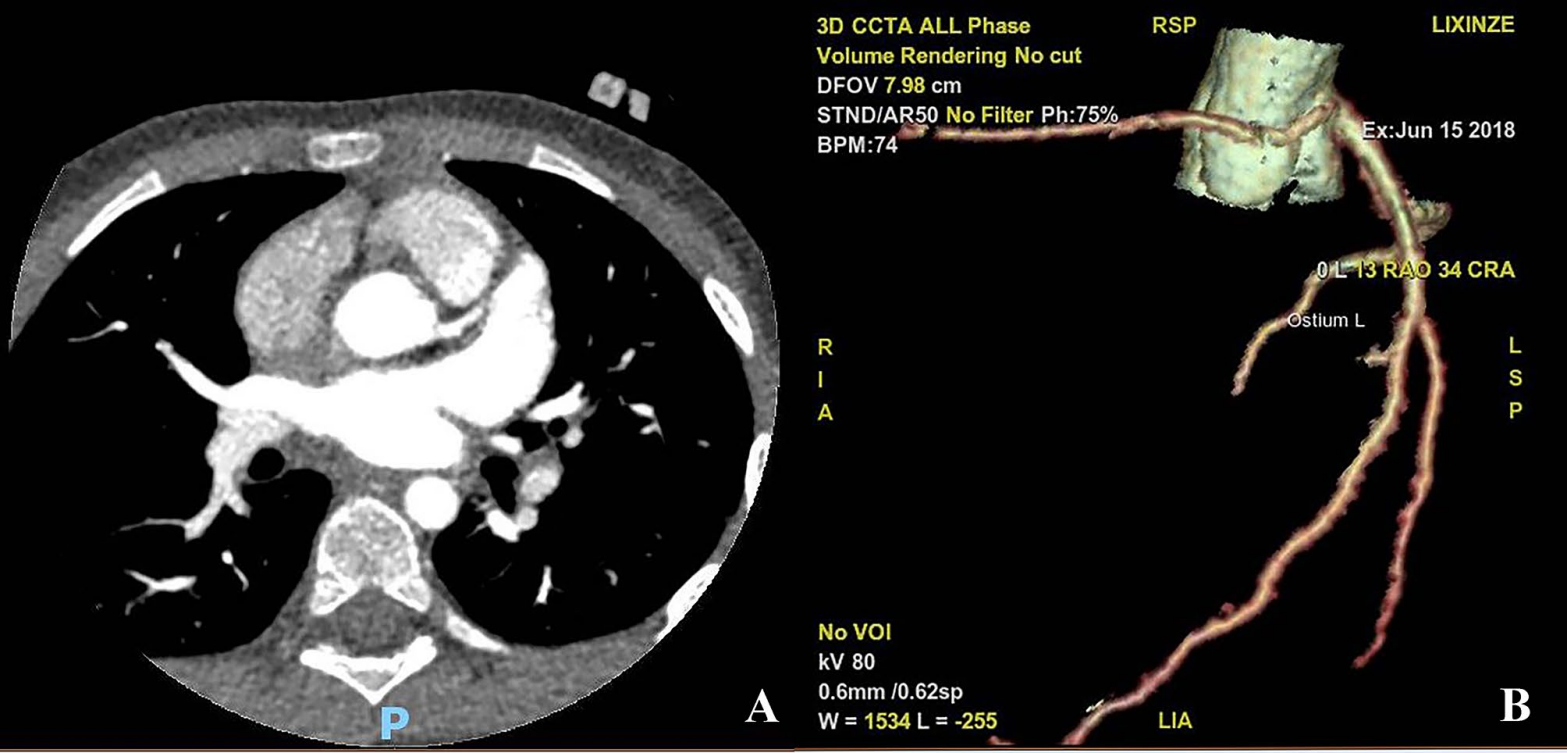
CCTA images in patients with ARCA (**A**, **B**). The right coronary artery originates from the left coronary sinus, and no coronary artery is seen in the right coronary sinus

**Fig. 2:**
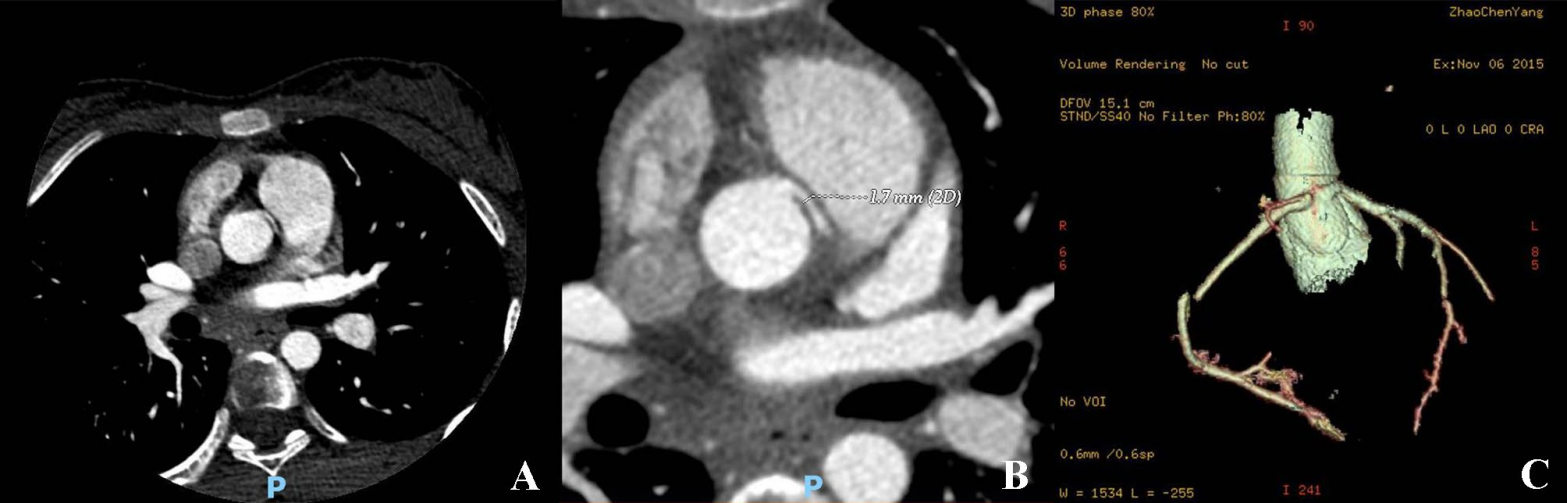
CCTA images in patients with ARCA (**A**–**C**). The left coronary artery originates from the right coronary sinus, and its proximal end runs between the ascending aorta and the right wall of the pulmonary artery (**A**). The proximal end of the left coronary artery is markedly narrow, with a diameter of approximately 1.7 mm (**B**)

A total of 69 children were diagnosed with AAOCA by CTCA, including 34 males (49.3%) and 35 females (50.7%), with a mean age of 8.89 ± 4.40 years. Of these, 10 (14.5%) presented with ALCA, 57 (82.6%) with ARCA, and two (2.9%) had a coronary artery originating from the non-coronary sinus (Figs. 1 and 2). Before the diagnosis was confirmed using CTCA, only 4 (5.8%) cases of AAOCA (all cases of ARCA) were identified by TTE; 59 patients (85.5%) were misdiagnosed or suspected to have myocarditis due to the evidence of myocardial injury or myocardial ischemia based on patients’ symptoms and test results. The follow-up period after discharge ranged from 2 months to 9 years.

### Clinical manifestations

Nineteen (27.5%) children were asymptomatic; 35 (50.7%) presented with minor symptoms such as chest tightness, chest pain, and fatigue; 13 (18.8%) had severe symptoms such as syncope; and 2 (2.9%) had atypical symptoms such as paroxysmal crying.

### Clinical characteristics of different AAOCA types

There were no statistically significant differences in sex, clinical manifestations, positive rate of myocardial damage markers, ECG, TTE, or proportion of high-risk anatomical features among the three groups (ALCA, ARCA, and coronary artery originating from the non-coronary sinus), as shown in Table 2.

**Table 2:** Clinical characteristics of different anomalous coronary artery types

		ALCA (n = 10)	ARCA (n = 57)	Coronary artery originating from the non-coronary sinus (n = 2)	χ^2^	p Value
Gender	Female	6 (60.0)	29 (50.9)	0 (0.0)	2.047	0.457^c^
	Male	4 (40.0)	28 (49.1)	2 (100.0)		
Clinical Manifestations	Asymptomatic	4 (40.0)	15 (26.3)	0 (0.0)	3.656	0.870^c^
	Minor symptoms	4 (40.0)	29 (50.9)	2 (100.0)		
	Severe symptoms	2 (20.0)	11 (19.3)	0 (0.0)		
	Atypical symptoms	0 (0.0)	2 (3.5)	0 (0.0)		
Myocardial damage markers	Positive	2 (20.0)	17 (29.8)	0 (0.0)	0.735	0.850^c^
	Negative	8 (80.0)	40 (70.2)	2 (100.0)		
ECG^a^	ST-T changes/pathological Q waves	8 (80.0)	27 (47.4)	2 (100.0)	7.045	0.497^c^
	Atrioventricular block/sinoatrial block	2 (20.0)	7 (12.3)	0 (0.0)		
	Sinus bradycardia/sinus arrest/sinoatrial block	0 (0.0)	12 (21.1)	0 (0.0)		
	Sinus tachycardia	0 (0.0)	4 (7.0)	0 (0.0)		
	Atrial premature beats/ventricular premature beats	0 (0.0)	7 (12.3)	0 (0.0)		
TTE^b^	Normal	7 (70.0)	52 (91.2)	2 (100.0)	3.699	0.141^c^
	Cardiac enlargement/decreased cardiac function	3 (30.0)	5 (8.8)	0 (0.0)		
High-risk anatomy	Without	4 (40.0)	20 (35.1)	2 (100.0)	3.055	0.204^c^
	With	6 (60.0)	37 (64.9)	0 (0.0)		

Values are expressed as N (%)

*ALCA* Anomalous origin of the left coronary artery from the right coronary artery sinus, *ARCA* anomalous origin of the right coronary artery from the left coronary artery sinus, *ECG* Electrocardiographic, *TTE* Transthoracic echocardiography

^a^Note that some patients have more than one ECG abnormality

^b^Note that some patients have cardiac enlargement with LVEF of ≤ 55%

^c^Calculated using Fisher’s exact test

### Clinical characteristics of children by age group

A comparison of clinical characteristics for different age groups showed a higher proportion of asymptomatic children in the infant and preschool groups (p < 0.001), and more atypical symptoms in infants. There were no significant differences in AAOCA type, positive myocardial damage markers, ECG, TTE, or proportion of high-risk anatomical features among children of different ages (Table 3).

**Table 3:** Clinical characteristics of children with anomalous aortic origin of a coronary artery at different ages

		Infant (n = 9)	Preschool (n = 11)	School age (n = 49)	χ^2^	p Value
Gender	Female	3 (33.3)	6 (54.5)	26 (53.1)	1.264	0.566^c^
	Male	6 (66.7)	5 (45.5)	23 (46.9)		
Clinical Manifestations	Asymptomatic	6 (66.7)	7 (63.6)	6 (12.2)	29.417	< 0.001^c^
	Minor symptoms	1 (11.1)	2 (18.2)	32 (65.3)		
	Severe symptoms	0 (0)	2 (18.2)	11 (22.4)		
	Atypical symptoms	2 (22.2)	0 (0.0)	0 (0.0)		
Anomalous coronary artery types	ALCA	2 (22.2)	0 (0.0)	8 (16.3)	3.025	0.525^c^
	ARCA	7 (77.8)	11 (100.0)	39 (79.6)		
	Coronary artery originating from the non-coronary sinus	0 (0.0)	0 (0.0)	2 (4.1)		
Myocardial damage markers	Positive	5 (55.6)	4 (36.4)	10 (20.4)	5.111	0.070^c^
	Negative	4 (44.4)	7 (63.6)	39 (79.6)		
ECG^a^	ST-T changes	3 (33.3)	7 (63.6)	27 (55.1)	6.003	0.613^c^
	Atrioventricular block	2 (22.2)	1 (9.1)	6 (12.2)		
	Sinus bradycardia	1 (11.1)	2 (18.2)	9 (18.4)		
	Sinus tachycardia	2 (22.2)	0 (0.0)	2 (4.1)		
	Sinoatrial block	1 (11.1)	1 (9.1)	5 (10.2)		
TTE^b^	Normal	6 (66.7)	11 (100.0)	44 (89.8)	4.537	0.071^c^
	Cardiac enlargement/decreased cardiac function	3 (33.3)	0 (0.0)	5 (10.2)		
High-risk anatomy	Without	5 (55.6)	7 (63.6)	14 (28.6)	6.013	0.052^c^
	With	4 (44.4)	4 (36.4)	35 (71.4)		

Values are expressed as N (%)

*ALCA* Anomalous origin of the left coronary artery from the right coronary artery sinus, *ARCA* anomalous origin of the right coronary artery from the left coronary artery sinus, *ECG* Electrocardiographic, *TTE* Transthoracic echocardiography

^a^Note that some patients have more than one ECG abnormality

^b^Note that some patients have cardiac enlargement with LVEF of ≤ 55%

^c^Calculated using Fisher’s exact test

### High-risk anatomical features and clinical symptom severity

The low-risk anatomy group included 26 (37.7%) patients, and the high-risk anatomy group included 43 (62.3%) patients. Inter-arterial course, orifice stenosis/slit-like orifice, and acute take-off angle were found in 37, 4, and 2 patients, respectively; no patient had an intra-mural course. Participants with high-risk anatomical features were more likely to have severe clinical symptoms and were prone to cardiac syncope (p < 0.05) (Tables 4 and 5).

**Table 4:** Relationship between high-risk anatomical features and symptom severity

	Asymptomatic	Minor symptoms	Severe symptoms	Atypical symptoms	χ^2^	p Value
High-risk anatomy	9 (20.9)	22 (51.2)	12 (27.9)	0 (0.0)	9.878	0.012^a^
Low-risk anatomy	10 (38.5)	13 (50.0)	1 (3.8)	2 (7.7)		

Values expressed as N (%)

^a^Calculated using Fisher’s exact test

**Table 5:** Break-up of different high-risk features frequency

**High-risk anatomy**	43 (62.3%)
**Inter-arterial course**	37 (53.6%)
**Orifice stenosis/slit-like orifice**	4 (5.8%)
**Acute take-off angle**	2 (2.9%)
**intra-mural course**	0

Values expressed as N (%)

### Treatment and prognosis

All children were treated with sodium creatine phosphate and coenzyme Q10 tablets if there was evidence of myocardial injury (symptoms and test results). Captopril was administered 0.5–1 mg/(kg·d) orally, twice a day, to children diagnosed with cardiac enlargement, until heart size returned to normal. If tachycardia occurred, metoprolol was administered 0.5–1 mg/(kg·d) orally, twice a day, to control the heart rate and increase coronary blood supply. Digoxin at a dose of 3–6 µg/(kg·d) orally, twice a day, was added for children with cardiac insufficiency. Five cases (7.2%) of suspected severe myocarditis were treated with methylprednisolone at a dose of 2–10 mg/(kg·d) combined with gamma globulin at 2 g/kg before the diagnosis of AAOCA and were discontinued after the diagnosis was made. Two patients (2.9%) with an ALCA and an inter-arterial course underwent coronary unroofing. All patients were successfully treated and discharged, and no cardiovascular events occurred during follow-up.

## Discussion

AAOCA is a congenital abnormality of the coronary artery origin that is commonly associated with SCD in adolescents, during exercise. Due to high accuracy in detecting abnormal coronary orifice and high-risk anatomy, CTCA is the main technique used to diagnose AAOCA in China [4]. The prevalence of AAOCA, which ranges from 0.27% to 2.26% in CTCA-evaluated cases [5], varies due to differences in population, inclusion criteria, and diagnostic methods. ARCA appear to be more common than ALCA [6], and coronary arteries originating from the non-coronary sinus are extremely rare, discussed only in case reports [7]. In our study, 57 patients with ARCA and 10 patients with ALCA were found, which is consistent with previous studies and the demographic characteristics of AAOCA [8–11]. Two patients (2.9%) had coronary arteries originating from non-coronary sinuses. This high prevalence may be explained by the small amount of data and the different methodologies used, as we only included children who were admitted to our hospital for cardiac symptoms or abnormal examinations. Krupiński et al. [12] used CTCA as the diagnostic method and demonstrated that ARCA was more likely to be complicated with high-risk anatomical features such as a slit-like orifice, inter-arterial course, and intra-mural courses in adult populations. Cheezum et al. [5] also showed that the frequency of inter-arterial ALCA was lower than that of inter-arterial ARCA. In this study, the proportion of children with high-risk anatomical features was similar regardless of AAOCA type, which may be related to differences in the population and diagnostic methods.

The clinical manifestations of AAOCA in children vary from asymptomatic to chest tightness, chest pain, palpitations, fatigue, and even cardiac syncope. In our study, the proportion of asymptomatic children in the infant and preschool groups was high. And symptoms in the infant group when present, were more atypical, which may lead to missed diagnosis AAOCA in children at young ages; however, myocardial ischaemia, as shown by ECG, TTE, or myocardial damage markers, can aid diagnosis. In patients with evidence of myocardial ischaemia and suspected AAOCA, CTCA should be performed after the condition has stabilised, to avoid misdiagnosis of myocarditis. We believe, coronary reserve with increased cardiac output during vigorous activity is limited in patients with AAOCA. Therefore, patients in the school age group presented symptoms more frequently than patients in the preschool or infant groups. Silvana et al. [13] evaluated 201 children with AAOCA and found that older age at diagnosis was a predictor of high-risk (high-risk anatomy and symptoms or evidence of myocardial ischaemia). For AAOCA patients with an inter-arterial course, the tension of the aorta and pulmonary artery increases during exercise, squeezing the coronary artery and reducing blood flow, which may result in myocardial ischaemia, angina pectoris, and SCD.

The AHA/ACC guidelines and previous studies generally suggest that ALCA is associated with a higher risk of SCD [2, 5, 14, 15]. The annual probability of SCD in athletes due to ALCA and ARCA is 0.35% (0.08%–0.9%) and 0.02% (0.0035%–0.06%), respectively [16]. However, recent studies have linked SCD to high-risk anatomy rather than AAOCA type. Finocchiaro et al. [17] found that the ARCA and ALCA with an inter-arterial course were the most common anatomical variants recognised in patients with SCD at the British Cardiac Pathology Centre. SCD usually occurs during exercise in patients with ALCA, and at rest in patients with ARCA. The nine cases of SCD during exercise reported by Kurosu et al. [15] were all AAOCA with inter-arterial courses and acute take-off angles.

Cardiac symptoms, especially syncope, may be precursors of SCD. Finocchiaro et al. [17] found that 37% of patients had cardiac symptoms (syncope, chest pain, and fatigue) before SCD, with syncope being the most common symptom. Kurosu et al. [15] reported several cases where SCD in children occurred during or immediately after exercise, including two patients with syncope followed by sudden death and one patient with chest pain. In a risk assessment conducted by Molossi et al. [13], the presence of an intra-mural course and syncope on exertion were predictors of high-risk lesions. None of the children included in this study developed SCD, but 2 in 10 cases of ALCA (20%) and 11 in 57 cases of ARCA (19.3%) presented with syncope, with no significant difference. There were also no significant differences in clinical characteristics between the ALCA, ARCA, and coronary arteries originating from the non-coronary sinus groups. However, we found that children with high-risk anatomy were more likely to experience syncope than those without (p < 0.05), which is consistent with previous studies [4, 5, 9].

The pathological mechanisms underlying the relationship between high-risk anatomy and clinical manifestations remain unclear. SCD and cardiac symptoms are often associated with exercise, which can stimulate myocardial contraction and dilate the aorta, thereby pressuring the inter-arterial or intra-mural coronary arteries, leading to insufficient coronary blood supply, myocardial ischaemia, and chest pain. Persistent myocardial ischaemia can also lead to decreased cardiac ejection fraction by causing arrhythmia and myocardial hyposystole, resulting in hypoperfusion of the brain and syncope. In addition, coronary artery torsion during exercise can lead to a decreased take-off angle and increased orifice stenosis, resulting in ischaemia and related symptoms [18]. In our study, eight (11.6%) children had left cardiac enlargement or decreased cardiac function (CTCA was performed after they were stabilized), and six of them had ST-elevated myocardial infarction. Myocardial infarction, as one of the manifestations of myocardial ischaemia, is common in patients with AAOCA [10]. This also indicates that AAOCA can lead to myocardial ischaemia and associated symptoms. Forty-three (62.3%) patients had high-risk anatomy, including 37 (53.6%) inter-arterial course, 4 (5.8%) orifice stenosis/slit-like orifice, and 2 (2.9%) acute take-off angle; no patient had an intra-mural course (Table 5). As patients with high-risk anatomical features were more likely to have myocardial ischaemia and lead to severe clinical manifestations, surgery is preferred, especially for those with chest pain or syncope and cardiac enlargement or decreased cardiac function.

Based on the pathological mechanisms underlying AAOCA-induced myocardial ischaemia, exercise restriction and drug therapy may be effective. Mery et al. [16] created two separate decision analysis models for ARCA and ALCA and compared three strategies (observation, exercise restriction, and surgery); surgery was deemed preferential in a small percentage of the population model and the majority favoured observation alone in patients aged ≤ 20 years, whereas exercise restriction was a suboptimal strategy for all age groups. However, long-term medical treatment and exercise restrictions in children can lead to emotional problems and cardiovascular diseases. Therefore, we selected patients for surgery according to the guidelines of the American Association for Thoracic Surgery [19]. The surgery was performed in 1) individuals with AAOCA and symptoms of ischaemic chest pain or syncope suspected to be caused by ventricular arrhythmias or a history of aborted SCD; 2) patients with ALCA and inter-arterial course (even in asymptomatic cases). However, many parents are concerned about the safety of surgery and choose to treat their children conservatively. Therefore, among the 69 children with AAOCA in our study, conservative treatment was the main strategy, metoprolol was administered to control the heart rate and increase coronary blood supply; only two (2.9%) patients with ALCA and inter-arterial course underwent coronary unroofing. No cardiovascular events occurred during hospitalization and follow-up. Due to myocardial scarring, incomplete unroofing, and coronary distortion with unroofing, high-risk anatomy may remains after surgery, myocardial ischaemic symptoms and SCD can occur [20, 21]. For these patients, β-blockers and other drugs are recommended postoperatively and long-term prognosis remains variable [22].

This study had a few limitations. First, the high-risk anatomical features included in our study were mainly inter-arterial course; other features (such as intra-mural course and orifice stenosis) may be unrelated to the patients’ symptoms [23]. Second, this was a single-centre retrospective study, which may result in low data volume and data collection bias. Therefore, large-scale multicentre studies are required to validate our findings.

In conclusion, there were no significant differences in the proportion of high-risk anatomical features and clinical manifestations among children with different AAOCA types. However, younger children were more likely to be asymptomatic and symptom severity may be related to high-risk anatomy. The pathological mechanism may involve myocardial ischaemia caused by aggravation of high-risk anatomy due to coronary artery compression and torsion, during exercise. Therefore, AAOCA should be considered in children with chest pain and syncope, especially those with exercise-related seizures; CTCA should be performed to identify high-risk anatomies and, when found, they should be treated accordingly.

## Data Availability

All data generated or analyzed during this study are included in this published article and the authors will supply the relevant data in response to reasonable requests.

## Abbreviations

AAOCA: Anomalous aortic origin of the coronary artery
ACC: American College of Cardiology
AHA: American Heart Association
ALCA: Anomalous origin of the left coronary artery
ARCA: Anomalous origin of the right coronary artery
BNP: Brain natriuretic peptide
CK-MB: Creatine kinase myocardial band
CTCA: Computerised tomography coronary angiography
ECG: Electrocardiogram
LVEF: Left ventricular ejection fraction
NT-proBNP: N-terminal pro-B-type natriuretic peptide
SCD: Sudden cardiac death
TnI: Troponin I
TTE: Transthoracic echocardiography
